# Circulating betatrophin/ANGPTL8 levels correlate with body fat distribution in individuals with normal glucose tolerance but not those with glucose disorders

**DOI:** 10.1186/s12902-020-0531-8

**Published:** 2020-04-16

**Authors:** Jing Zheng, Juan Liu, Beverly S. Hong, Weijian Ke, Minmin Huang, Yanbing Li

**Affiliations:** 1 Department of Endocrinology, The First Affiliated Hospital of Sun Yat-sen University, Guangzhou, China; 2 Department of Endocrinology, The Affiliated Hospital of Guizhou Medical University, Guiyang, China

**Keywords:** Betatrophin/ANGPTL8, Body fat distribution, Normal glucose tolerance

## Abstract

**Background:**

The relationship between betatrophin/ANGPTL8 and obesity has been investigated using body mass index (BMI); however, since BMI reflects overall adiposity rather than body fat distribution, it remains unclear whether fat deposition in different areas of the body affects betatrophin expression. Here, we investigated the correlation between circulating betatrophin levels and body fat distribution in patients with different glucose tolerance.

**Methods:**

We performed a cross-sectional study in 128 participants with impaired glucose tolerance (IGT; *n* = 64) or normal glucose tolerance (NGT; *n* = 64). Circulating betatrophin levels were detected by enzyme-linked immunosorbent assay (ELISA). Body fat distribution (subcutaneous, visceral, and limb fat) was measured by magnetic resonance imaging (MRI) and a body fat meter.

**Results:**

After controlling for age, sex, and BMI, betatrophin was correlated positively with visceral adipose tissue-to-subcutaneous adipose tissue ratio (VAT/SAT ratio; *r* = 0.339, *p* = 0.009) and negatively with body fat ratio (BFR; *r* = − 0.275, *p* = 0.035), left lower limb fat ratio (LLR; *r* = − 0.330, *p* = 0.011), and right lower limb fat ratio (RLR; *r* = − 0.288, *p* = 0.027) in the NGT group, with these correlations remaining after controlling for triglycerides. VAT/SAT ratio (standardized *β* = 0.419, *p* = 0.001) was independently associated with serum betatrophin levels; however, betatrophin was not associated with body fat distribution variables in the IGT group.

**Conclusions:**

Circulating betatrophin levels correlated positively with VAT/SAT ratio and negatively with lower limb fat, but not with subcutaneous or upper limb fat, in individuals with normal glucose tolerance. Thus, betatrophin may be a potential biomarker for body fat distribution in individuals without glucose disorders.

## Background

Betatrophin, also known as ANGPTL8, lipasin, C19orf80, TD26, or RIFL, is a member of the angiopoietin-like protein family that is expressed in liver and adipose tissue [1–3]. Multiple members of this protein family are closely related to obesity and obesity-related metabolic diseases: ﻿ANGPTL3, ANGPTL4, and ANGPTL6 can directly regulate lipid, glucose, and energy metabolism without exerting angiogenic effects [4]. Betatrophin, a nutritionally-regulated factor, also involves in the pathophysiology of lipid metabolism [5–7], presenting with that betatrophin is an important regulator of plasma triglycerides (TGs) [5, 6, 8]. Serum TG levels are reduced in betatrophin-null mice [6] and dramatically increased in betatrophin-overexpressing mice [5, 7]. Betatrophin also plays an important role in lipid storage in adipocytes. In 3T3-L1 adipocytes, the knockdown of betatrophin during adipogenesis quantitatively and significantly decreases neutral lipid levels, while recombinant betatrophin increases intracellular TG levels [8]. Furthermore, a recent study showed that *Angptl8* antisense oligonucleotides protect fat-fed mice against hepatic steatosis and insulin resistance by promoting adipose lipoprotein lipase (LPL) activity and inhibiting ectopic lipid accumulation [9].

Recently, an increasing number of studies have focused on the relationship between betatrophin and obesity; however, their results have been controversial [10–13]. Jia et al. demonstrated that serum betatrophin levels are significantly elevated in overweight patients but not in those with obesity [13], whereas another study showed that betatrophin levels are higher in obese individuals than in the non-obese population [11]. Lee [14] and Ren [12] found that betatrophin levels are higher in both overweight and obese subjects. Betatrophin levels were also observed to be decreased in morbidly obese individuals (BMI > 40 kg/m^2^) but not change in obese individuals (BMI 30–40 kg/m^2^) [15]. Some studies have explored the effect of weight change on betatrophin, presenting with inconsistent results. A study reported that serum betatrophin levels decrease after diet-related weight loss [16] while another noted that only surgery-induced weight loss increases blood betatrophin levels [17].

Why were the inconsistent results observed in these clinical studies? One possible explanation is that only BMI was used to evaluate the degree of obesity of the participants in these studies. As we know, although it is widely used as a proxy to estimate overall adiposity and total fat mass in clinical studies, BMI either cannot accurately distinguish between fat and lean mass, or reflect the distribution of body fat. We speculate that betatrophin could be synthesized only in adipose tissue of some parts of the body, rather than all adipose tissue, which may be the reason of inconsistent results regarding the relationship between BMI and betatrophin. Our previous study suggested that betatrophin levels are positively correlated with ﻿hepatic lipid deposition independently of obesity [18]. Von Loeffelholz et al. found that omental fat betatrophin mRNA expression is significantly higher in obese patients with ﻿liver steatosis and insulin resistance than in BMI-matched insulin-sensitive subjects [3]. However, whether or not fat distribution plays a role in the relationship between obesity and betatrophin is still unclear. Herein, we performed a cross-sectional study to explore the correlation between blood betatrophin levels and body fat distribution in patients with different glucose tolerance status.

## Methods

### Study population

A total of 128 subjects were recruited from the Department of Endocrinology of the First Affiliated Hospital of Sun Yat-sen University in a nationwide multi-center investigation known as the “Early Identification and Intervention Techniques of Metabolic Syndrome Study” between October 2012 and November 2013. The participants either had impaired glucose tolerance (IGT; *n* = 64) or were age- and sex-matched subjects with normal glucose tolerance (NGT; *n* = 64). IGT diagnoses were based on diagnostic criteria issued by the American Diabetes Association (ADA) in 2012 [19]. Subjects were excluded from the study based on the following criteria: those treated with oral antidiabetic, hypolipidemic, and/or antihypertensive agents, and those with active hepatitis, renal or liver dysfunction, congestive heart failure,﻿ or other known major diseases. The study was approved by the Ethics Committee Board of the First Affiliated Hospital of Sun Yat-sen University. All participants received oral and written information about the study and provided written informed consent.

### Anthropometric measurements and biochemical evaluations

Blood samples were collected from an antecubital vein in the morning after an overnight fast to analyze glucose, insulin, and betatrophin levels and lipid profiles. Blood samples were also collected 120 min after glucose ingestion as part of the 75 g oral glucose tolerance test (OGTT) to measure plasma glucose and serum insulin levels. HbA1c was measured using high-pressure liquid chromatography. Serum betatrophin levels were determined using a commercially available human enzyme-linked immunosorbent assay (ELISA) kit (cat no. E11644h; Wuhan Eiaab Science, Wuhan, China). Samples were measured in duplicate according to the manufacturer’s protocol. β cell function was assessed by homeostasis model assessment of β cell function (HOMA-β) [20]. Insulin resistance was estimated by index of homeostasis model assessment of insulin resistance (HOMA-IR) [20], quantitative insulin sensitivity check index (QUICKI) [21] and the Matsuda insulin sensitivity index (Matsuda ﻿ISI) [21]

### Measurement of body fat distribution

#### Measurement of abdominal subcutaneous and visceral fat

Participants were examined using abdominal coil magnetic resonance imaging (MRI; 3-Tesla whole-body scanner; SIEMENS 3.0 T MAGNETOM Verio; Siemens Healthcare Sector, Germany), as described previously [18, 22]. The same radiologist performed all abdominal MRI scans. Abdominal subcutaneous adipose tissue (SAT) and visceral adipose tissue (VAT) were evaluated by calculating the abdominal subcutaneous fat area (SFA) and visceral fat area (VFA) separately. The boundary for the SFA region of interest (ROI) was defined between the abdominal skin contour and the outer margin of the abdominal wall muscles, while the VFA ROI was defined between the inner margin of the abdominal wall muscles and the anterior border of the spinal column.

#### Measurement of body and limb fat

The body fat ratio (BFR), upper limb fat ratios, and lower limb fat (including gluteal fat) ratios were measured using a body fat meter (Tanita MC-180, Tokyo, Japan). Subjects wearing a single garment were instructed to stand naturally on the body fat meter with bare feet, making sure that their feet and hands made close contact with the plate electrode. Values were read from a computer connected to the body fat meter.

### Statistical analysis

All statistical analyses were performed using SPSS version 21.0 (SPSS, Chicago, Illinois). Data were presented as the mean ± SD (for normally distributed variables) or the median (25th and 75th percentiles; for non-normally distributed variables). Data that were not normally distributed were logarithmically transformed for statistical analysis. Differences among groups were analyzed using analysis of variance (ANOVA) followed by Fisher’s least significant difference (LSD) test. The Kolmogorov-Smirnov test was used to analyze non-normally distributed data. Differences in gender distribution were analyzed using χ^2^ analysis. Correlation coefficients were analyzed using Spearman’s (non-normally distributed data) or Pearson’s (normally distributed data) rank correlation. To elucidate the independent relationship between betatrophin and clinical parameters, we selected betatrophin as a dependent variable and other clinical parameters as the independent variables to build a multiple linear stepwise regression equation. Only variables that were significantly (*P* < 0.05) related to betatrophin by Spearman or Pearson correlation analyses were entered into the multiple linear stepwise regression analysis. *P* values of < 0.05 were considered statistically significant.

## Results

### Circulating betatrophin levels do not differ in patients with IGT and NGT

The baseline clinical characteristics of the study participants are listed in Table 1. The betatrophin concentrations and body fat distribution indices did not differ between the NGT and IGT groups. The IGT group had higher 2 h-PG and alanine aminotransferase (ALT) levels (*p* < 0.05) and significantly lower BMI and Matsuda ﻿ISI than the NGT group (*p* < 0.05); however, no differences were observed between the other anthropometric and biochemical variables in the two groups.

**Table 1:** Clinical and biochemical characteristics of the study subjects from different groups

Variables	NGT (***n*** = 64)	IGT (***n*** = 64)	***P*** value
Age^a^	52.98 ± 6.39	53.58 ± 6.82	0.357
Sex, male/female (%)	32/32	32/32	1.000
BMI (kg/m^2^)^a^	25.15 ± 2.25	24.18 ± 2.62*	0.033
WHR	0.88 ± 0.06	0.88 ± 0.06	0.697
SBP (mmHg)	122.88 ± 14.04	125.05 ± 15.06	0.607
DBP (mmHg)	75.59 ± 11.89	73.09 ± 10.68	0.986
ALT (U/L)^a^	17.00 (14.00–23.00)	25.00 (16.25–29.75)*	0.003
AST (U/L)^a^	22.00 (18.00–24.00)	23.00 (20.00–26.75)	0.058
GGT (U/L)^a^	25.00 (18.00–36.75)	29.00 (21.00–43.00)	0.212
HbA1c (%)	5.63 ± 0.49	5.69 ± 0.43	0.280
TC (mmol/L)	5.27 ± 0.86	5.25 ± 0.95	0.611
TG (mmol/L)^a^	1.45 ± 0.87	1.80 ± 1.84	0.062
LDL cholesterol (mmol/L)	3.46 ± 0.86	3.37 ± 0.88	0.962
HDL cholesterol (mmol/L)^a^	1.28 ± 0.38	1.23 ± 0.37	0.618
SAT (cm^2^)^a^	170.75 (139.35–216.08)	152.55 (126.98–200.88)	0.120
VAT (cm^2^)^a^	86.97 (57.43–111.90)	94.22 (69.67–120.93)	0.363
VAT/SAT^a^	0.47 (0.33–0.76)	0.52 (0.44–0.74)	0.084
BFR (%)^a^	25.30 (19.50–32.20)	26.30 (21.10–31.10)	0.940
LUR (%)	25.00 ± 8.07	24.61 ± 7.76	0.305
RUR (%)	24.48 ± 7.94	23.88 ± 7.71	0.396
LLR (%)	26.95 ± 9.56	26.25 ± 10.19	0.280
RLR (%)	26.92 ± 9.51	26.02 ± 10.47	0.169
FPG (mmol/L)^a^	5.20 (4.83–5.60)	5.10 (4.80–5.50)	0.460
2 h-PG (mmol/L)^a^	6.10 (5.00–6.90)	8.90 (8.40–9.60)*	0.000
FINS (μU/mL)^a^	7.96 (5.22–9.83)	8.64 (5.97–11.33)	0.157
HOMA-IR^a^	1.74 (1.19–2.41)	2.03 (1.38–2.61)	0.263
HOMA-β^a^	87.00 (64.55–119.05)	107.39 (70.54–146.16)	0.087
QUICKI^a^	0.35 (0.33–0.37)	0.34 (0.33–0.36)	0.230
Matsuda ﻿ISI	6.12 ± 3.36	4.72 ± 2.91*	0.013
Betatrophin (pg/mL)^a^	708.52 (562.72–895.82)	729.72 (543.09–1022.67)	0.443

*BMI* Body mass index, *WHR* Waist-to-hip ratio, *SBP* Systolic blood pressure, *DBP* Diastolic blood pressure, *ALT* Alanine aminotransferase, *AST* Aspartate aminotransferase, *GGT* Gamma-glutamyl transpeptidase, *TC* Total cholesterol, *TG* Triglyceride, *LDL* Low-density lipoprotein cholesterol, *HDL* High-density lipoprotein cholesterol, *SAT* Subcutaneous adipose tissue, *VAT* Visceral adipose tissue, *VAT/SAT* Ratio visceral adipose tissue-to-subcutaneous adipose tissue ratio, *BFR* Body fat ratio, *LUR* Left upper limb fat ratio, *RUR* Right upper limb fat ratio, *LLR* Left lower limb fat ratio, *RLR* Right lower limb fat ratio, *FPG* Fasting plasma glucose**,** *2 h-PG* 2 h-plasma glucose during oral glucose tolerance test﻿**,** *FINS* Fasting blood insulin, *HOMA*-*β* Homeostasis model of β cell function, *HOMA-IR* Homeostasis Model of insulin resistance, *QUICKI* Quantitative insulin sensitivity check index, *Matsuda* ISI Matsuda insulin sensitivity index. Data are presented as the mean ± SD (normally distributed) or median (25th and 75th percentiles; non-normally distributed). Differences between groups were analyzed by ANOVA followed by the LSD test. Gender distribution differences were analyzed by χ2 analysis. * *p* < 0.05 vs. the NGT group. ^a^Non-normally distributed data were transformed logarithmically for statistical analysis

### Betatrophin levels correlate with body fat distribution indicators in patients with NGT

**Fig. 1:**
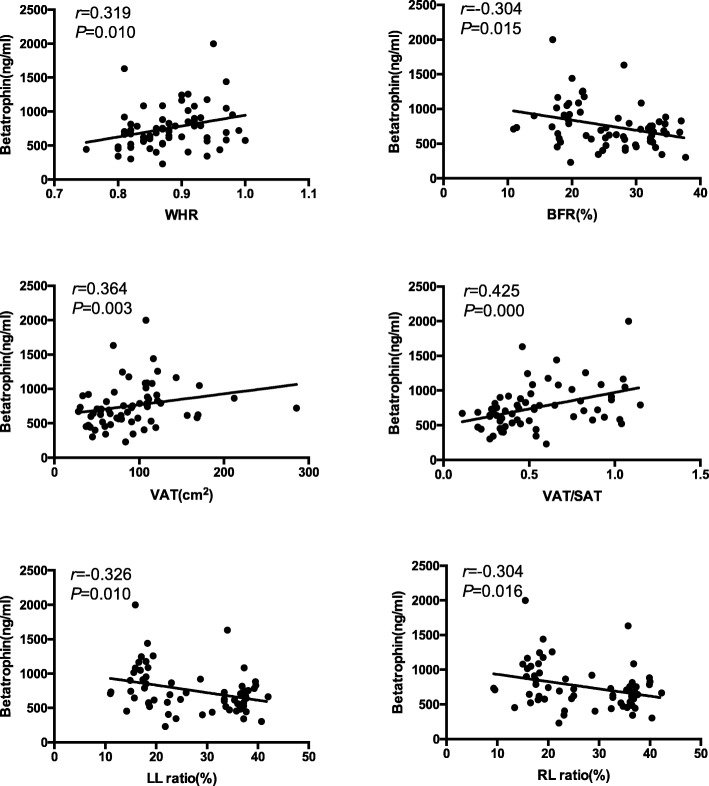
Plasma betatrophin concentrations correlated positively with WHR (*r* = 0.319, *p* = 0.010), BFR (*r* = -0.304, *p* = 0.015), VAT (*r* = 0.364, *p* = 0.003), VAT/SAT (*r* = 0.425, *p* < 0.001), LL ratio (*r* = − 0.326, *p* = 0.010), and RL ratio (*r* = − 0.304, *p* = 0.016) in the NGT group

Correlation analysis revealed that betatrophin levels correlated positively with the WHR (*r* = 0.319, *p* = 0.010), VAT (*r* = 0.364, *p* = 0.003), and VAT/SAT ratio (*r* = 0.425, *p* < 0.001) and negatively with the BFR (*r* = − 0.304, *p* = 0.015), LLR (*r* = − 0.326, *p* = 0.010), and RLR (*r* = − 0.304, *p* = 0.016) in the NGT group (Table 2 and Fig. 1). After controlling for age, sex, and BMI, betatrophin levels correlated positively with the VAT/SAT ratio (*r* = 0.339, *p* = 0.009) and negatively with the BFR (*r* = − 0.275, *p* = 0.035), LLR (*r* = − 0.330, *p* = 0.011), and RLR (*r* = − 0.288, *p* = 0.027; Table 2). ﻿When also controlling for TG, the correlations between betatrophin levels and these variables (VAT/SAT ratio, BFR, LLR, and RLR) remained but were slightly attenuated (Table 2); however, no associations were detected between betatrophin levels and any body fat distribution variables in the IGT group (Table 3).

**Table 2:** Correlation analysis of body fat distribution and betatrophin levels in the NGT group

	Betatrophin		Betatrophin (age, sex, and BMI adjusted)		Betatrophin (age, sex, BMI, and TG adjusted)	
	***r***	***P*** value	Partial ***r***	***P*** value	Partial ***r***	***P*** value
WHR	0.319	0.010*	0.175	0.185	0.191	0.151
SAT	− 0.192	0.128	− 0.189	0.151	− 0.199	0.135
VAT	0.364	0.003*	0.239	0.069	0.244	0.064
VAT/SAT	0.425	0.000*	0.339	0.009*	0.355	0.006*
BFR (%)	−0.304	0.015*	−0.275	0.035*	−0.269	0.041*
LUR (%)	−0.236	0.065	0.044	0.740	0.040	0.767
RUR (%)	−0.171	0.184	0.163	0.217	0.156	0.244
LLR (%)	−0.326	0.010*	−0.330	0.011*	−0.324	0.013*
RLR (%)	−0.304	0.016*	−0.288	0.027*	−0.281	0.033*

*WHR* Waist-to-hip ratio, *SAT* Subcutaneous adipose tissue, *VAT* Visceral adipose tissue, *VAT/SAT* Ratio visceral adipose tissue-to-subcutaneous adipose tissue ratio, *BFR* Body fat ratio, *LUR* Left upper limb fat ratio, *RUR* Right upper limb fat ratio, *LLR* Left lower limb fat ratio, *RLR* Right lower limb fat ratio. Statistical significance from Pearson’s (normally distributed data) or Spearman’s (non-normally distributed data) correlation tests. *P values < 0.05 were considered statistically significant

**Table 3:** Correlation analysis of body fat distribution and betatrophin levels in the IGT group

	Betatrophin		Betatrophin (age, sex, and BMI adjusted)		Betatrophin (age, sex, BMI and TG adjusted)	
	***r***	***P*** value	Partial ***r***	***P*** value	Partial ***r***	***P*** value
WHR	0.003	0.984	−0.022	0.866	−0.015	0.911
SAT	−0.012	0.923	−0.054	0.680	−0.034	0.796
VAT	−0.055	0.666	−0.054	0.682	−0.046	0.731
VAT/SAT	−0.024	0.850	0.000	0.998	−0.009	0.944
BFR (%)	0.002	0.987	−0.070	0.593	−0.064	0.629
LUR (%)	−0.001	0.991	0.096	0.464	0.114	0.390
RUR (%)	0.026	0.842	0.183	0.162	0.192	0.145
LLR (%)	0.058	0.652	0.018	0.889	0.022	0.866
RLR (%)	0.036	0.780	−0.004	0.977	0.001	0.993

*WHR* Waist-to-hip ratio, *SAT* Subcutaneous adipose tissue, *VAT* Visceral adipose tissue, *VAT/SAT* Ratio visceral adipose tissue-to-subcutaneous adipose tissue ratio, *BFR* Body fat ratio, *LUR* Left upper limb fat ratio, *RUR* Right upper limb fat ratio, *LLR* Left lower limb fat ratio, *RLR* Right lower limb fat ratio. Statistical significance from Pearson’s (normally distributed data) or Spearman’s (non-normally distributed data) correlation tests. *P values < 0.05 were considered statistically significant

### Betatrophin correlates independently with the VAT/SAT ratio

To determine whether serum betatrophin levels were independently associated with body fat distribution indices, we performed multiple stepwise linear regression analysis. This revealed that the VAT/SAT ratio (standardized *β* = 0.419, *p* = 0.001) was independently associated with serum betatrophin levels in subjects with NGT (Table 4).

**Table 4:** Multiple stepwise regression analysis of betatrophin levels and the variables of body fat distribution in the NGT group

Independent variable	Standardized ***β***	***t*** statistic	***P*** value
VAT/SAT	0.419	3.572	0.001*
BFR (%)	−0.153	−1.160	0.251
LLR (%)	−0.131	−0.926	0.358
RLR (%)	−0.106	−0.744	0.460

*VAT/SAT* Ratio visceral adipose tissue-to-subcutaneous adipose tissue ratio, *BFR* Body fat ratio, *LLR* Left lower limb fat ratio, *RLR* Right lower limb fat ratio. *P values < 0.05 were considered statistically significant

## Discussion

Most of previous studies have focused on the association between betatrophin and obesity or BMI. In the past 2 years, researchers have begun to turn their attention to the relationship between betatrophin and body fat distribution. Kriebel et al. sampled visceral and subcutaneous fat from patients with or without hepatic steatosis to detect betatrophin mRNA expression, with the results that betatrophin mRNA levels were higher in the VAT than the SAT in both groups [3]. Another study found that circulating betatrophin levels have an inverse relationship with SAT expression in lean and obese patients with and without T2DM, suggesting that the local effect of betatrophin on adipose tissue is independent of obesity [23]. These two studies also confirmed that betatrophin is not specifically secreted by liver tissues, as previously reported by Zhang [5], but is also secreted by visceral and subcutaneous fat. In this study, we used a noninvasive approach to explore the relationship between betatrophin and body fat distribution in patients with different glucose tolerance status. We interestingly found that betatrophin levels correlated positively with VAT/SAT ratio which is consistent with the results of Kriebel [3], and negatively with lower body adiposity including lower limb and gluteal fat in NGT subjects, but not IGT subjects, indicating that betatrophin levels could be closely associated with body fat distribution in NGT subjects.

Which mechanism plays a role in the emergence of this result? ﻿As is well known, the contribution of various adipose tissue deposits varies for the risk of metabolic disease [24–26]. Visceral adiposity is regarded as be more closed with metabolic diseases such as hypertension, diabetes, and dyslipidemia compared to other tissue fat deposits [26]. Thus, the VAT/SAT ratio which is a metric of relative body fat composition has been proposed to be an independent predictor of death and coronary events [25, 27]. In addition, visceral obesity has also been defined as a predictor of nonalcoholic fatty liver disease (NAFLD) [28]. Unlike visceral adiposity, lower body subcutaneous adiposity which accumulates in the thighs and hips is thought to be metabolically protective [25]. Many studies reported that the accumulation and infiltration of macrophages in adipose tissue to active inflammation could be the underlying mechanism. Obesity-associated adipose tissue inflammation varies between individuals, possibly due to depot-specific differences [8]. ﻿In mice and humans, VAT contains a higher percent of proinflammatory M1 macrophages and CD4 Th1 T-cells than in SAT [29]. Pinnick et al. provided an evidence ﻿to support an increased macrophage presence in abdominal SAT, whereas no corresponding enrichment was observed in gluteal SAT. Ejarque et al. reported that macrophages can express and secrete ANGPTL8 in their preliminary experiments [30]. Therefore, we speculate that the higher the VAT or VAT/SAT, the more macrophages would accumulate and infiltrate in the adipose tissue, and the more betatrophin would be secreted. The opposite trend could be observed when lower limb fat increased. The speculation needs to be verified in further study.

Betatrophin has recently emerged as an indicator of metabolic disorders, with two separate case-control studies finding that betatrophin levels are ﻿elevated in subjects with metabolic syndrome and hypertension [31, 32]. A Chinese study of non-diabetic individuals found that circulating full-length betatrophin levels are an independent risk factor for coronary artery disease (CAD) and are positively associated with its severity [33]. The homogeneity in the correlation between VAT/SAT ratio, betatrophin, and metabolic disorders may be due to their close association, as observed in this study.

It is therefore reasonable to speculate that the association between abnormal body fat distribution and cardiovascular and metabolic diseases may be partially mediated by betatrophin. Indeed, a growing body of evidence has suggested that body fat distribution is closely related to the inflammatory state of the body [25–27, 34]. Moreover, VAT accumulation and a higher VAT/SAT ratio may also be associated with increased chronic low-grade systemic inflammation, which could further increase betatrophin synthesis [25, 26, 31]. Correspondingly, elevated betatrophin levels may contribute toward the pathogenesis of dyslipidemia, which is one of the most important risk factors for CAD, while in vitro and in vivo studies have suggested that ﻿betatrophin could aggravate hypertriglyceridemia by promoting the ability of ANGPTL3 to bind and inhibit LPL [5, 6, 35]. Clinical studies have also confirmed that betatrophin levels are significantly and positively related to TG and LDL-C levels and inversely related to HDL-C levels in children and patients with diabetes [1, 7, 36, 37] [38]. In addition, betatrophin is positively correlated with age [36], liver fat content [18], and blood pressure [32], all of which are independent risk factors for atherosclerosis and may contribute to the occurrence and development of CAD.

In addition, our findings may explain why previous clinical studies have yielded inconsistent and even opposite betatrophin levels in obese or overweight people [10, 11, 13, 39, 40]. This is likely due to the different baseline characteristics of the populations recruited in these clinical trials, including age, sex, lifestyle, genetics, and gene-environment interactions that can influence body fat distribution [41]. In this study, we also found that correlations between betatrophin and body fat distribution indices only existed in the NGT group, not the IGT group. We speculate that the different glucose metabolic states of the patients could affect their inflammatory state, since increased inflammatory cytokine levels affect betatrophin synthesis [14, 42–44], accordingly the relationship between betatrophin and body fat distribution cannot be observed. It should be noted that the hypothesis requires further elucidation. In addition, other factors such as the presence of cardiovascular disease, hypertension, and dyslipidemia, which are common in IGT group, could affect the expression of this protein, as demonstrated in previous studies [32, 33]. Previous animal experiments have confirmed that betatrophin was a pivotal regulator of plasma triglycerides. Serum triglycerides levels are reduced in ANGPTL8-null mice [13] and increased dramatically in ANGPTL8 overexpressing mice [5, 7]. However, as are inconsistent with the results of animal experiments, many clinical studies [10, 12, 13, 36, 37] including the present study did not find any relationship between betatrophin and triglycerides. So, the adjustment of TG levels cannot affect the results of the nonsignificant relationship between betatrophin and fat body distribution. After adjusting for age, sex, BMI, the results were still the same. The possible reason is that there is no statistical difference in these variables between NGT group and IGT group.

Our study has several limitations. Firstly, food ingestion greatly affects betatrophin levels; however, our analyses were based on single blood betatrophin measurements obtained under fasting conditions, which may not reflect betatrophin levels over time. Secondly, the cross-sectional design of this study allowed us to observe the correlation between VAT/SAT ratio and betatrophin levels but cannot prove causality between the two variables. Thirdly, since no patients with impaired fasting glycemia were enrolled in this study due to its relatively low prevalence, our findings do not fully reflect the metabolic characteristics of prediabetes; however, this does not affect the conclusions drawn from the NGT population. Lastly, the ethic differences in the expression or plasma concentrations of betatrophin should be considered. However, as we know, no related study has ever been published. The present study was carried out just in Chinese population, the results of which cannot directly extend to other ethic population.

In summary, the findings of this study could provide new insights into the possible contribution of betatrophin to the pathogenesis of obesity. We demonstrated that betatrophin levels are correlated with body fat distribution in individuals with NGT, showing a significant positive correlation with VAT/SAT ratio and negative correlation with lower body fat. The gold standard methods for assessing body fat distribution include CT and MRI, which allow the amount of adipose tissue deposited in particular depots to be accurately evaluated [24]; however, their time-consuming nature and high cost limit their clinical applications [41]. The findings of this study suggest that betatrophin could be a favorable indicator that reflects body fat distribution during the normal stage of glucose intolerance and could be a simple and reliable risk assessment surrogate for CAD and metabolic disease in clinical practice. However, the mechanisms via which this protein affects ectopic body fat distribution remain unclear and further studies are warranted.

## Conclusion

Circulating betatrophin levels correlated positively with VAT/SAT ratio and negatively with lower limb fat in individuals with NGT. Thus, betatrophin may be a biomarker for body fat distribution in individuals without glucose disorders.

## Data Availability

The datasets are available from the corresponding author on reasonable request (Yanbing Li, Email: easd04lyb@126.com).

## Abbreviations

BMI: Body mass index
TG: Triglyceride
IGT: Impaired glucose tolerance
NGT: Normal glucose tolerance
SBP: Systolic blood pressure
DBP: Diastolic blood pressure
ALT: Alanine aminotransferase
AST: Aspartate aminotransferase
GGT: Gamma-glutamyl transpeptidase
TC: Total cholesterol
TG: Triglyceride
LDL: Low-density lipoprotein cholesterol
HDL: High-density lipoprotein cholesterol
Matsuda ISI: Matsuda insulin sensitivity index
ADA: American Diabetes Association
OGTT: Oral glucose tolerance test
ELISA: Enzyme-linked immunosorbent assay
HOMA-β: Homeostasis model of β cell function
HOMA-IR: Homeostasis model of insulin resistance
QUICKI: Quantitative insulin sensitivity check index
MRI: Magnetic resonance imaging
SAT: Subcutaneous adipose tissue
VAT: Visceral adipose tissue
VAT/SAT ratio: Visceral adipose tissue-to-subcutaneous adipose tissue ratio
LUR: Left upper limb fat ratio
RUR: Right upper limb fat ratio
LLR: Left lower limb fat ratio
RLR: Right lower limb fat ratio
SFA: Subcutaneous fat area
VFA: Visceral fat area
ROI: Region of interest
BFR: Body fat ratio
NAFLD: Nonalcoholic fatty liver disease
CAD: Coronary artery disease
LPL: Lipoprotein lipase
LSD: Least significant difference
ANOVA: Analysis of variance
